# EVAR solution for acute thrombosis of abdominal aortic aneurysm: A case report and literature review

**DOI:** 10.1097/MD.0000000000043941

**Published:** 2025-08-08

**Authors:** Xiping Xu, Shumeng Yang, Jihua Shi, Tao Qin, Yubin Li

**Affiliations:** a Department of Vascular Surgery, Linyi People’s Hospital, Shandong Second Medical University, Linyi, Shandong, China.

**Keywords:** abdominal aortic aneurysm, acute thrombosis, balloon thrombectomy, endovascular aneurysm repair

## Abstract

**Rationale:**

Acute thrombosis of an abdominal aortic aneurysm (ATAAA) is a rare but catastrophic complication of abdominal aortic aneurysm. For inherently high-risk ATAAA, a more invasive open surgical approach is associated with increased mortality.

**Patient concerns:**

A 68-year-old male presented with sudden-onset severe back and lower limb pain persisting for over 9 hours. Physical examination revealed a pulsatile abdominal mass and bilateral lower limb ischemia.

**Diagnoses:**

Computed tomography angiography revealed a 4.5 cm infrarenal abdominal aortic aneurysm with acute thrombosis extending into both common iliac arteries, confirming the diagnosis of ATAAA.

**Interventions:**

The proximal neck anatomy of the aneurysm was suitable for endovascular aneurysm repair (EVAR); accordingly, balloon thrombectomy followed by EVAR was performed.

**Outcomes:**

Complete revascularization was achieved. The patient was discharged on postoperative day 4 and remained asymptomatic at the 2-year follow-up.

**Lessons:**

Only 5 cases of ATAAA treated with an endovascular approach have been reported. Although further validation is needed, EVAR shows promise as a safe and effective treatment for selected cases, potentially reducing the high mortality rate associated with this condition.

## 1. Introduction

Acute thrombosis of an abdominal aortic aneurysm (ATAAA) is a rare but catastrophic complication of abdominal aortic aneurysm (AAA). Since its first description by Shumacker in 1959,^[1]^ most reported cases have been managed with open surgery, including in situ aortic reconstruction and extra-anatomic bypass, with a mortality rate of 46%–59% reported in the limited available literature.^[2,3]^ Endovascular aneurysm repair (EVAR) offers potential advantages in reducing this high mortality rate owing to its minimally invasive nature. To date, only 5 cases of ATAAA treated with an endovascular approach have been reported^[4–8]^; herein, we present an additional case that was successfully managed with EVAR.

## 2. Case report

A 68-year-old male was transferred to our emergency department with sudden-onset severe back and lower limb pain for over 9 hours. Additional signs and symptoms included lower-limb pallor, coolness, paresthesia, numbness, and progressive paralysis, accompanied by mild nausea. He reported a transient episode of back pain while lifting heavy objects approximately 20 hours prior to admission. His only comorbidity was a 40-year smoking history, with no previous history of intermittent claudication.

**Figure 1.**
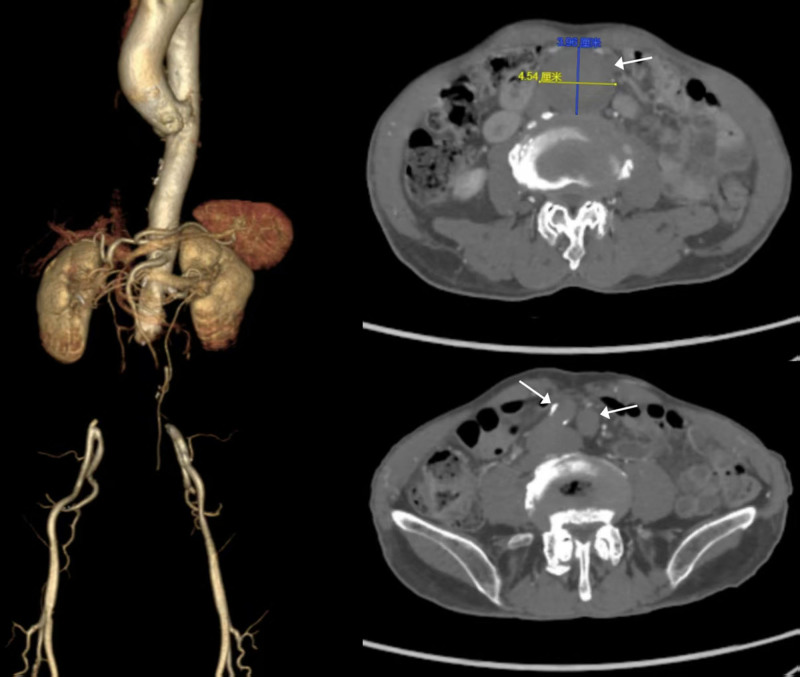
Preoperative CTA showed acute thrombosis of a 4.54 cm infrarenal AAA and both common iliac arteries (white arrows). The proximal neck of the AAA was patent and suitable for EVAR. AAA = abdominal aortic aneurysm, CTA = computed tomography angiography, EVAR = endovascular aneurysm repair.

Physical examination revealed a tender pulsatile abdominal mass, but the bilateral femoral and distal pulses were nonpalpable. Electrocardiography revealed sinus bradycardia (53 beats/minute). Echocardiography demonstrated moderate mitral and aortic regurgitation without an intracardiac thrombus. Computed tomography angiography revealed a 4.5 cm infrarenal AAA with acute thrombosis extending from the aorta to both common iliac arteries, which was suitable for EVAR (Fig. 1). Laboratory studies showed elevated D-dimer (6.62 μg/mL [ref: 0–0.5 μg/mL]) and creatine kinase (519.0 U/L [ref: 38.0–174.0 U/L]), with creatinine below the reference range (59 μmol/L [ref: 62–115 μmol/L]).

**Figure 2.**
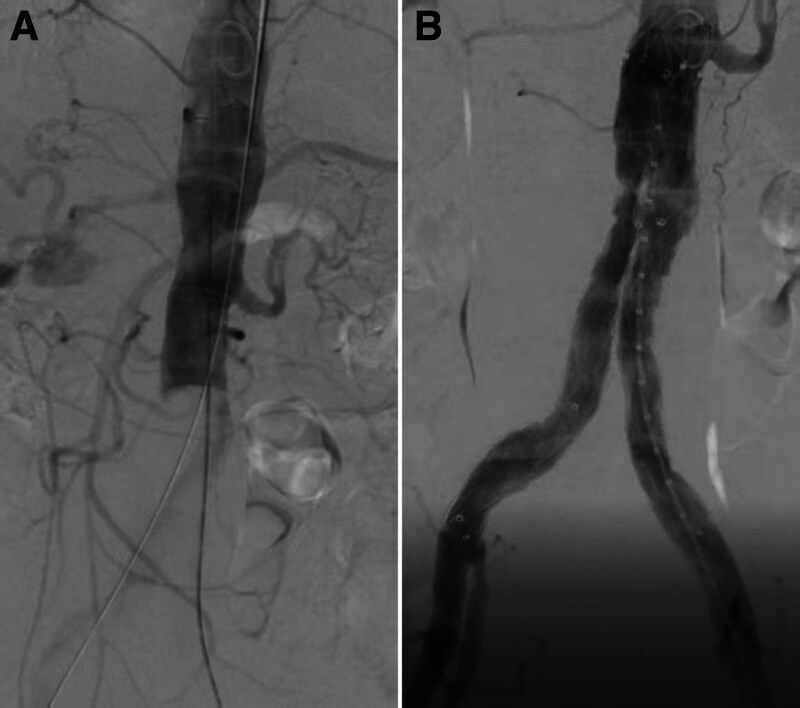
Aortography before (A) and after (B) EVAR. The stent-graft was well-positioned without apparent stenosis or endoleaks. EVAR = endovascular aneurysm repair.

After obtaining written informed consent, the patient was urgently transferred to a hybrid operating room equipped with a high-resolution imaging system (Artis Zeego, Siemens, Germany). Under general anesthesia, both common femoral arteries (CFAs) were surgically exposed and cross-clamped distally. After systemic heparinization, the CFAs were transversely incised to a length of approximately 5 mm. Balloon thrombectomy of the aorta and bilateral iliac arteries was performed using a 0.035-inch guidewire (Terumo, Tokyo, Japan) and a 6-French Fogarty Thru-Lumen Embolectomy Catheter (Edwards Lifesciences, Irvine). Some fresh thrombi were removed, allowing the guidewire to reach the neck of the aneurysm. However, subsequent aortography via a 5-French Pigtail Catheter (Cordis, Miami Lakes) showed failure of infrarenal aortic recanalization (Fig. 2A).

A decision was made to use a stent-graft to recanalize the infrarenal aorta and exclude the AAA. After exchanging the guidewire for an Amplatz Ultra-Stiff guidewire (Cook Medical, Bloomington), the trunk-ipsilateral limb of an Excluder AAA Endoprosthesis (Percutek Therapeutics, Beijing, China) was advanced from the right side and deployed below the left renal artery. The endoprosthesis was unwrapped and expanded smoothly without predilatation, and cannulation of the contralateral limb was achieved effortlessly. To mitigate the risk of endoleaks, both limbs of the stent-graft were extended distally to the bifurcations of the common iliac arteries, with balloon molding (Boston Scientific, Marlborough) performed at the proximal and distal anchoring segments and the overlapping junctions. Completion aortography demonstrated a well-positioned stent-graft without apparent endoleaks or stenosis, with preservation of the right internal iliac artery (Fig. 2B).

The entire procedure was performed under transient femoral artery clamping to prevent distal embolization and to allow flushing of any thrombus or debris. After repairing the CFAs, distal pulses became palpable. The operation time was 3 hours, and the time from admission to revascularization was approximately 5 hours. No intraoperative complications occurred. The patient’s postoperative course was uneventful; he began ambulating on postoperative day 4 and was discharged later that day. At the 2-year telephone follow-up, the patient was engaged in farming activities.

## 3. Discussion

ATAAA has a reported incidence of 0.7%–2.8% among all surgically managed AAAs, often resulting in catastrophic consequences.^[2]^ Proposed etiologies include occlusive iliac artery disease, hypercoagulable states, cardiogenic thromboembolism, and mural thrombus dislodgment.^[2,3]^ The most common symptoms are the classic “6 Ps” of acute limb ischemia (pain, pallor, pulselessness, paresthesia, paralysis, and poikilothermia), occasionally accompanied by back pain; differential diagnoses include saddle embolism, aortic dissection, and ruptured AAA.^[4,6,7]^ In the present case, the patient suffered from transient back pain while lifting heavy objects approximately 20 hours before admission, which potentially triggered mural thrombus dislodgment. This dislodgment occluded the aneurysm outflow, leading to retrograde thrombosis.

In 1961, Jannetta and Roberts^[9]^ reported the first well-documented revascularization for ATAAA using a bifurcated Teflon graft. Since then, traditional open surgical repair involving aneurysmectomy and in situ aortic reconstruction has remained the preferred treatment option, providing definitive management of both the aneurysm and acute occlusive events. The less invasive extra-anatomic bypass is considered an alternative treatment for high-risk patients; however, thrombosed AAAs have been reported to carry a risk of delayed rupture.^[10–12]^ Therefore, an additional procedure of aortic ligation via the retroperitoneal approach may be necessary.

EVAR is widely used for AAAs, which has transformed the treatment approach for AAA in high-risk patients, offering lower operative mortality and morbidity rates compared to open surgery. However, the application of EVAR in ATAAA is still in its infancy; to date, only 5 cases of ATAAA treated with EVAR have been reported, with an encouraging 100% survival rate (Table 1).^[4–8]^ These 5 cases demonstrate that EVAR can be an effective treatment option for ATAAA, particularly in patients at high surgical risk or with severe comorbidities. Notably, the diameters of the AAAs in these cases ranged from 3.5 to 8.0 cm, with only one case meeting the criteria for intervention.^[4,13]^ Therefore, vigilance regarding the risk of acute thrombosis is warranted, even for small aneurysms. EVAR approaches were tailored to individual cases: femoral access was predominantly used, with brachial access employed adjunctively for occlusion balloon placement or guidewire traversal; stent-grafts were selected based on AAA morphology and the extent of thrombosis, including tube grafts, bifurcated grafts, and aorto-uni-iliac grafts.^[4,6,8]^ Adjunctive measures included systemic heparinization, distal femoral clamping, and sheath flushing to prevent distal embolization, with Fogarty balloon thrombectomy added in one case for extensive aortoiliac thrombus.^[5]^

**Table 1:** Reported cases of acute thrombosis of abdominal aortic aneurysm treated with endovascular aneurysm repair.

References	Age/sex	AAA size (cm)	Thrombosis extent	Treatment approach	Follow-up	Unique features
Robaldo et al^[4]^	89/M	8.0	AAA + bilateral CIA	Aorto-uni-iliac stent + femorofemoral bypass	3 months: small type IB endoleak	EVAR application in COVID-19 context
Uotani et al^[5]^	77/M	4.2	AAA + bilateral IIA/EIA	Fogarty balloon thrombectomy + EVAR	1 year: patency	First reported combination of EVAR and balloon thrombectomy
Pillai et al^[6]^	75/F	3.5	AAA (distal aorta patent)	Brachial access EVAR	1 year: asymptomatic	Emergency use of brachial access and peripheral stents
Terai et al^[7]^	89/M	5.0	AAA (aneurysmal wall ulcer, IMH)	Bilateral femoral access EVAR	None	EVAR for occlusion caused by intra-aneurysmal dissection
Kumar^[8]^	58/M	3.8	AAA (small flow to iliacs)	Aortic occlusion balloon + EVAR	10 months: asymptomatic	Early exploration of EVAR for ATAAA

AAA = abdominal aortic aneurysm, ATAAA = acute thrombosis of abdominal aortic aneurysm, CIA = common iliac artery, EIA = external iliac artery, EVAR = endovascular aneurysm repair, F = female, IIA = internal iliac artery, IMH = intramural hematoma, M = male.

In the present case, given the adequate proximal aneurysm neck and thrombosed aortoiliac arteries, EVAR combined with balloon thrombectomy was considered a viable option. Although repeated attempts at balloon thrombectomy failed to recanalize the infrarenal aorta, the unimpeded passage of the thrombectomy balloon catheter suggested favorable anatomical conditions for stent-graft deployment.

Compared with conventional EVAR, several considerations should be emphasized when performing EVAR for ATAAA. First, both sides are viable candidates for the main body access route, particularly in cases of bilateral iliac artery occlusions. Antegrade access or aorto-uni-iliac stent-grafting with femorofemoral bypass is another alternative when only one route is available. Second, type I endoleaks may be common due to thrombus-induced instability of the anchoring segments. Immediate post-EVAR aortography and early detection of endoleaks are mandatory to prevent further aneurysm enlargement or even delayed rupture. Finally, the patient should also be prepared for conversion to open surgery if needed during EVAR.

## 4. Conclusion

Our case further demonstrates that EVAR is feasible for ATAAA in anatomically suitable patients; however, vigilance against thrombus-related challenges remains necessary. Although there are insufficient cases to confirm its safety and efficacy, the successful outcomes suggest that EVAR should be considered to mitigate the high mortality rates associated with traditional open surgery.

## Author contributions

**Conceptualization:** Xiping Xu, Shumeng Yang, Jihua Shi, Tao Qin, Yubin Li.

**Investigation:** Xiping Xu, Shumeng Yang, Jihua Shi, Tao Qin, Yubin Li.

**Methodology:** Shumeng Yang, Jihua Shi, Tao Qin, Yubin Li.

**Project administration:** Yubin Li.

**Software:** Xiping Xu, Shumeng Yang.

**Supervision:** Yubin Li.

**Visualization:** Xiping Xu, Shumeng Yang, Jihua Shi, Tao Qin, Yubin Li.

**Writing – original draft:** Xiping Xu.

**Writing – review & editing:** Yubin Li.
